# Mathematical Modeling to Predict COVID-19 Infection and Vaccination Trends

**DOI:** 10.3390/jcm11061737

**Published:** 2022-03-21

**Authors:** Bogdan Doroftei, Ovidiu-Dumitru Ilie, Nicoleta Anton, Sergiu-Ioan Timofte, Ciprian Ilea

**Affiliations:** 1 Faculty of Medicine, University of Medicine and Pharmacy “Grigore T. Popa”, University Street, No. 16, 700115 Iasi, Romania; bogdandoroftei@gmail.com (B.D.); anton.nicoleta1@umfiasi.ro (N.A.); cilea1979@yahoo.com (C.I.); 2 Department of Biology, Faculty of Biology, “Alexandru Ioan Cuza” University, Carol I Avenue, No. 20A, 700505 Iasi, Romania; sergiu.ioan.timofte@gmail.com

**Keywords:** COVID-19, SARS-CoV-2, Romania, doses, vaccination scheme, reactogenicity, ARIMA

## Abstract

Background: COVID-19 caused by the Severe Acute Respiratory Syndrome Coronavirus 2 placed the health systems around the entire world in a battle against the clock. While most of the existing studies aimed at forecasting the infections trends, our study focuses on vaccination trend(s). Material and methods: Based on these considerations, we used standard analyses and ARIMA modeling to predict possible scenarios in Romania, the second-lowest country regarding vaccinations from the entire European Union. Results: With approximately 16 million doses of vaccine against COVID-19 administered, 7,791,250 individuals had completed the vaccination scheme. From the total, 5,058,908 choose *Pfizer–BioNTech*, 399,327 *Moderna*, 419,037 *AstraZeneca*, and 1,913,978 *Johnson & Johnson*. With a cumulative 2147 local and 17,542 general adverse reactions, the most numerous were reported in recipients of *Pfizer–BioNTech* (1581 vs. 8451), followed by *AstraZeneca* (138 vs. 6033), *Moderna* (332 vs. 1936), and *Johnson & Johnson* (96 vs. 1122). On three distinct occasions have been reported >50,000 individuals who received the first or second dose of a vaccine and >30,000 of a booster dose in a single day. Due to high reactogenicity in case of AZD1222, and time of launching between the *Pfizer–BioNTech* and *Moderna* vaccine could be explained differences in terms doses administered. Furthermore, ARIMA(1,1,0), ARIMA(1,1,1), ARIMA(0,2,0), ARIMA(2,1,0), ARIMA(1,2,2), ARI-MA(2,2,2), ARIMA(0,2,2), ARIMA(2,2,2), ARIMA(1,1,2), ARIMA(2,2,2), ARIMA(2,1,1), ARIMA(2,2,1), and ARIMA (2,0,2) for all twelve months and in total fitted the best models. These were regarded according to the lowest MAPE, *p*-value (*p* < 0.05, *p* < 0.01, and *p* < 0.001) and through the Ljung–Box test (*p* < 0.05, *p* < 0.01, and *p* < 0.001) for autocorrelations. Conclusions: Statistical modeling and mathematical analyses are suitable not only for forecasting the infection trends but the course of a vaccination rate as well.

## 1. Introduction

Even though the number of human coronavirus infections per year is low [1], the current health crisis caused by the Severe Acute Respiratory Syndrome Coronavirus 2 (SARS-CoV-2) has placed each country worldwide in an unprecedented state of emergency [2]. Originally was theorized to be another low-level respiratory outbreak similar to the previous health crises [3,4,5,6] and pathophysiology analogies with SARS-CoV-2 [7].

It led initially to a relatively insignificant number of pneumonia cases [2] with an unknown etiology. Additional experiments brought new data and insight regarding the emergence of a novel beta-strain that belongs to the zoononic coronavirus (CoV) that is particularly virulent to humans [8]. Until the genome of this pathogen was fully sequenced, the number of cases confirmed already reached 15 with 1 fatality [9].

With fifty-nine confirmed cases at the beginning of 2020 [10], it became the main priority reflected by the associated mortality rate and high tropism towards the respiratory system [11]. COVID-19 has caused >280 million infections, and >5 million individuals passed away, from which more than 1.8 million infections and >58.00 deaths in Romania. The peak since the pandemic started in terms of infections and deaths was reached on 20th October with 18,863 cases of infections, and on 3 November with 591 deaths.

The need to model daily cases is essential to predict future directions. It is even more imperative for Eastern European countries such as Romania [12,13]. There are multiple reasons and limitations compared to Westernized countries [14,15]. Therefore, mathematical and statistical models became an integrated component within the current methodologies, dedicated to understanding the infectious and/or vaccination trend over a dedicated time interval [16]. Distinct approaches with relatively high accuracy in predicting trends failed because of randomness tendency of epidemics [17].

Systematically designed, approved, and distributed vaccines manufactured based on distinct procedures are already extensively discussed elsewhere [18,19,20,21] as a countermeasure to prophylactic strategies [22]. The first person vaccinated in Romania was a medical doctor with the candidate vaccine BNT162b1/BNT162b2 manufactured by *Pfizer–BioNTech* on 27 December 2020. On 4th February, the Romanian government approved and began using mRNA-1273 by *Moderna* vaccine, whereas AZD1222 (ChAdOx1) by *AstraZeneca* received approval 11 days later on 15 February. However, only on 4 May Ad26.COV2-S from *Johnson & Johnson* was available for the general population.

Such studies are crucial because of the low vaccination rate registered in middle-class countries. Presently, Romania has the second-lowest rate of vaccination among all European Union countries after Bulgaria. This subject caused a lot of controversies around individuals. Among the main factors that contribute to this generalized skepticism are of personal nature or beliefs.

Based on all these considerations, this article focuses on the number of doses administered depending on the manufacturer, those who completed or not the vaccination scheme, adverse reactions displayed by the recipients, and forecasts.

## 2. Materials and Methods

### 2.1. Data Collection and Parameters Analyzed

Data related to (1) the number of doses administered (dose 1, dose 2, and booster), (2) those that had or not completed the vaccination scheme, and (3) adverse reactions of local or general type were taken from the official website of the Romanian Government (https://vaccinare-covid.gov.ro/ (accessed on 28 December 2021)). We centralized all data by creating a time-series database using MS Excel for the following predetermined interval (27 December 2020–27 December 2021).

### 2.2. ARIMA

We divided the interval into short subdivisions with a forecast of 7 days. The error rate is dependent on the period forecasted based on our previous experience [23]. Equations, variants, model selection, and parameters are presented in former studies conducted by our team [23,24].

### 2.3. Statistical Analyses

Data analysis for three out of four parameters was carried out using Microsoft Excel 2010, whereas the software used for ARIMA modeling is STATGRAPHICS Centurion (18.1.14).

## 3. Results

**Figure 1:**
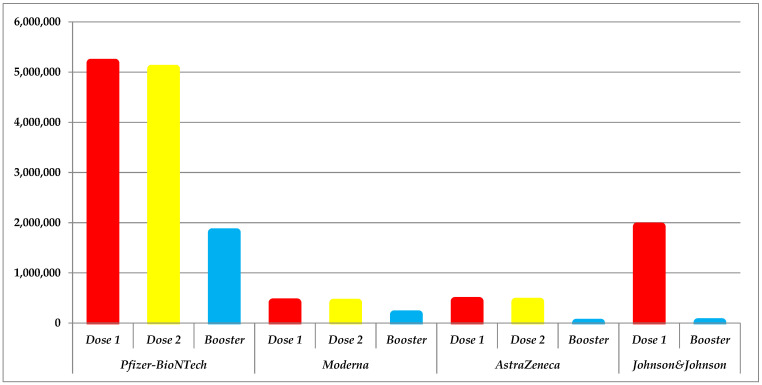
Graphic revealing trends for each dose depending on the manufacturer among Romanian individuals.

We observe that a significant percentage of the Romanian citizens were willing to receive the serum from *Pfizer–BioNTech*. Following the centralization and analysis of data, we noted a fluctuating trend that lasted several months before reaching the peak on 21st April 2021. With 55,643 (SE-724.01, SD-13,851.18, CI95%-1423.76) individuals immunized in a single day, we expected an increase since the second dose became available starting with 17 January 2021. Precisely, 11 May 2021 was the day in which was registered the second most numerous groups with 54,757 (SE-732.40, SD-13,603.80, CI95%-1440.55). Unfortunately, this trend gradually decreased again from May until mid-October. After this decline, the phase normalized with 2 successful waves of recipients and a maximum of 35,425 (SE-691.87, SD-6600.03, CI95%-1374.52) recipients of booster on 29 September 2021. From that point onwards, all three trends significantly decrease, this possibly having to do with the relaxation of restrictions in our country. Even though the technology behind manufacturing is the same as BNT162b1/BNT162b2, the *Moderna* vaccine did not benefit from the same interest among citizens. With figures suggesting that approximately 15.27% of recipients have chosen mRNA-1273 by comparison with *Pfizer–BioNTech*, on 5 February 2021, 8501 (SE-107.58, SD-1930.60, CI95%-211.66) people received the first dose. The situation remained the same also towards the introduction of the second dose on 12 February 2021. The highest number of individuals that attended a vaccination center was 8225 (SE-112.24, SD-1944.12, CI95%-220.88) on 5 March 2021. On 9 October 2021, a total of 21,951 (SE-232.45, SD-2217.51, CI95%-461.81) people receive a booster dose. Furthermore, the highest number of individuals vaccinated with AZD1222 on one day was on 26 February and 23 April last year. Despite the relatively high figures in both cases, 11,284 (SE-189.48, SD-3067.14, CI95%-373.12 and 10.685 (SE-203.60, SD-3067.57, CI95%-401.20), trends declined almost entirely in short-time. Two hundred and forty-five (0.05%) individuals decided to get the second dose of *AstraZeneca* vaccine and fewer (*n* = 4) for booster only in December if we refer to the overall situation. Not only the number of doses administered did not exceed 100, but there is also a gap between 2 November and 14 December 2021. As opposed to the rest by design and technology, *Johnson & Johnson* is the only available vaccine that requires a single dose. Similar to other trends, this was linear most of the time over the analyzed interval, with one exception on 27 October 2021 with 57,359 (SE-552.61, SD-8525.30, CI95%-1088.66). On 8 November 2021 started to be administered the second dose of Ad26.COV2-S according to WHO, CDC, and EMA directives. Thus, a maximum of 568 (SE-25.24, SD-178.48, CI95%-50.72) booster doses on 21 December 2021 were administered (Figure 1).

**Figure 2:**
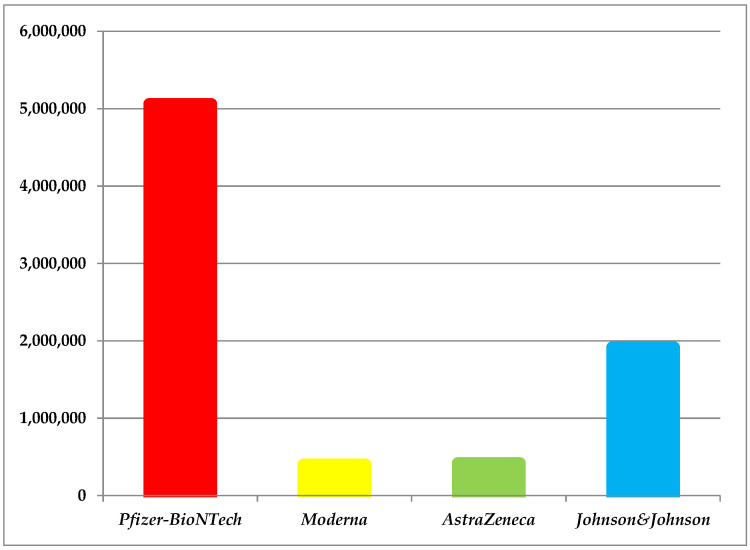
Graphic showing the total number of Romanian individuals who completed the vaccination scheme.

At the end of the analyzed interval, a total of 7,791,250 individuals were completely immunized. From this, 64.93%, *n* = 5.058.908 with BNT162b1/BNT162b2, and 24.59%, *n* = 1,913,978 with Ad26.COV2-S. Only a small percentage selected mRNA-1273 or ChAdOx1 (10.49% *n* = 818,364). As observed, both reached a plateau phase, this highlighting the human reluctance as a consequence of high reactogenicity. In this context, only 5.12%, *n* = 399,327 and 5.37%, *n* = 419,037 were attributed to the last two COVID-19 vaccines (Figure 2).

**Figure 3:**
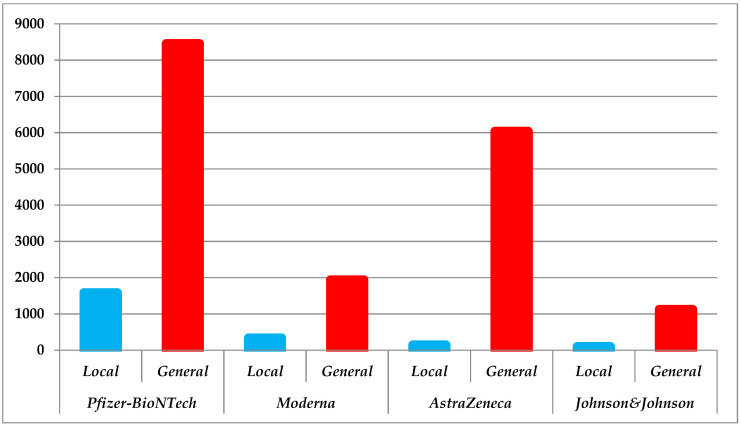
Graphic highlighting the total number of adverse reactions (local vs. general) reported in Romanian individuals.

There was a cumulative total of 2147 local adverse reactions and 17,542 general. Individually, 73.63%, *n* = 1581 were local (max = 56 on 5 January 2021) and 48.17%, *n* = 8451 (max = 170 on 6 February 2021) generalized in the case of BNT162b1/BNT162b2 followed by ChAdOx1 with 6.42%, *n* = 138 (max = 10 on 16 March 2021) and 34.39%, *n* = 6033 (max = 232 on 13th March 2021). Despite this, the Romanian citizens still opted for *Pfizer–BioNTech*. Through the prism of one critical argument might be explained this situation: the number of cases ≥100 on twenty-two different occasions, hence the lack of confidence in the serum from *AstraZeneca*. Continuing with this concept, 15.46%, *n* = 332 (max = 11 on 6 April 2021), whereas 11.03%, *n* = 1936 (max = 53 on 22 February 2021) of all adverse reactions were caused by *Moderna*. In more than eight months since its release, only 4.47% of the cases, *n* = 96 (max = 5 on 5 June 2021) led to local adverse reactions and 6.39%, *n* = 1122 (max = 45 on 26 October 2021) to those of generalized reported for *Johnson & Johnson* (Figure 3).

**Figure 4:**
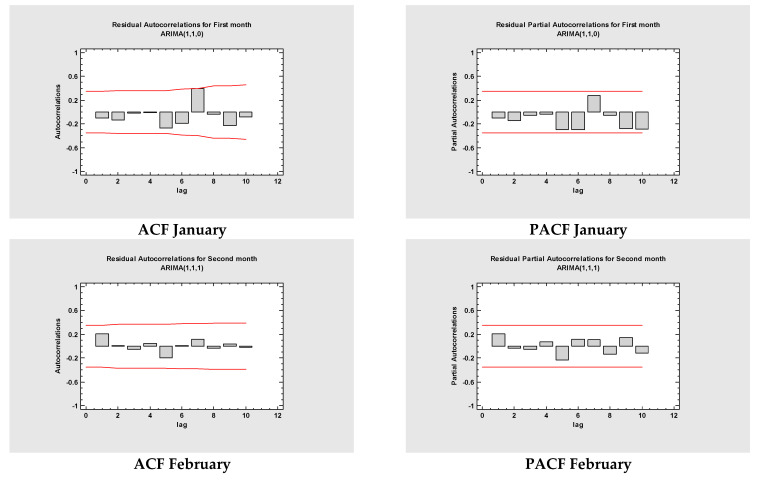

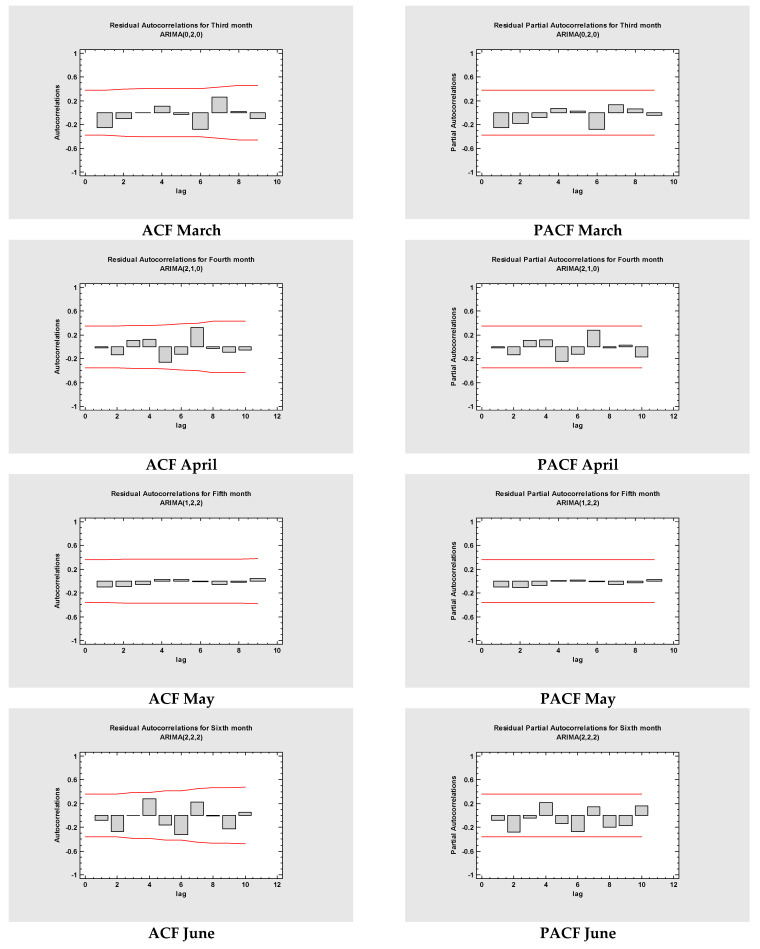

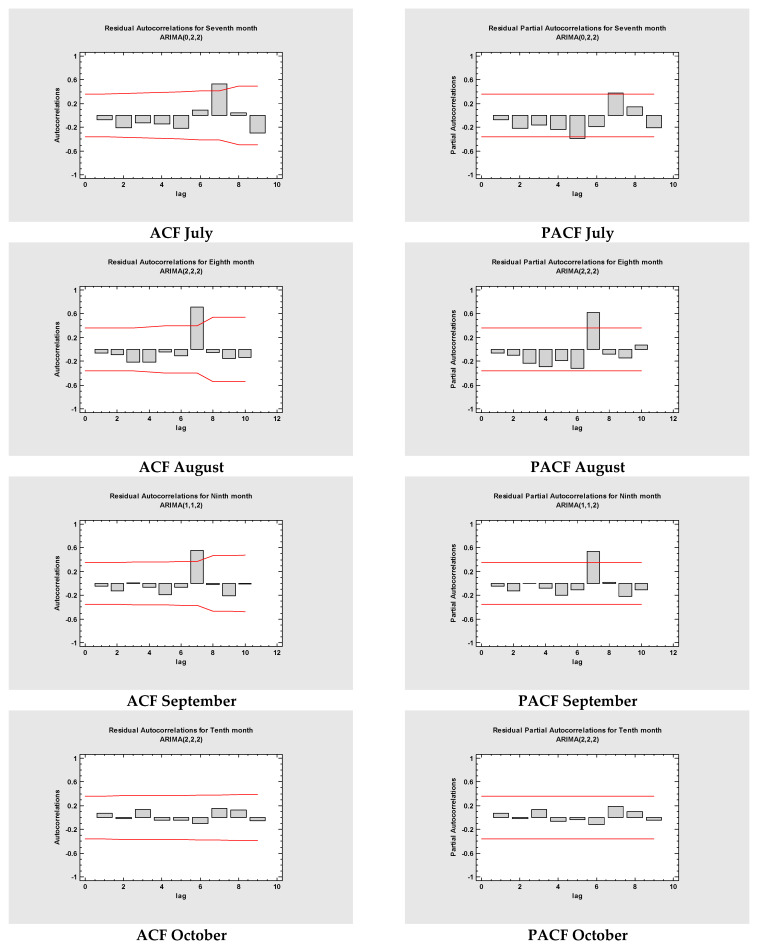

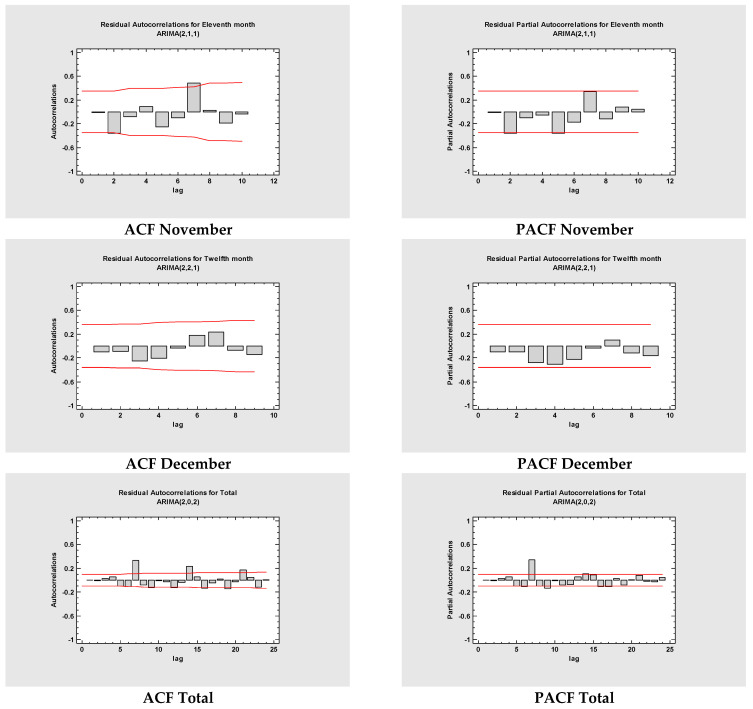
The estimated ACF and PACF graphs used to predict the vaccination trend against COVID-19 in Romania per each month and cumulative. In this figure (left and right) are displayed the associated plots for the estimated partial and autocorrelations between residuals at distinct lags. Specifically, the lag x, partial and autocorrelation, coefficients evaluate the affinity between the residuals at a time t and time (x − t) (for autocorrelation)/(x + t) (partial autocorrelations) at 95.0% probability to be close to 0. Distinct from autocorrelation is the condition that t + x accounts for the correlations at all lower lags, observation used to appreciate the order of autoregressive model where needed to fit the data. Valid for both functions if the probability at a specific for autocorrelation/particular for partial autocorrelation lag do not contain the estimated coefficient, indeed exists a statistically significant correlation at that lag at CI 95.0%.

According to Elevli et al. [25], to successfully create any ARIMA model, it must first meet four conditions and evaluate if a series of values are constant throughout the analyzed period. Autocorrelation Function (ACF), and Partial Autocorrelation Function (PACF) (Figure 4), are the time-series plots developed to evaluate the seasonality and stationarity. ACF is a metric that describes if the previous values are related to the next ones, whereas PACF determines the degree of correlation coefficient between variable and lag [26]. The performance of the model and misspecification detection is measured through the Bayesian information criterion of Schwarz (BIC), and Akaike information criteria expression (AIC) [27]. Straight lines points to the limit of two standard deviations, while the bars that cross the lines indicate statistically meaningful autocorrelations (Figure 4).

Performances of multiple models were generated and interpreted. MAPE with the lowest value per statistical analysis was regarded as the best model. Among all models, ARIMA(1,1,0), ARIMA(1,1,1), ARIMA(0,2,0), ARIMA (2,1,0), ARIMA(1,2,2), ARIMA(2,2,2), ARIMA(0,2,2), ARIMA(2,2,2), ARIMA(1,1,2), ARIMA(2,2,2), ARIMA(2,1,1), ARIMA(2,2,1), and ARIMA(2,0,2) were chosen for all twelve months and total. The fitted models are presented in Figure 4 and Table 1 and Table 2 with a minimum *MAPE_January_* = 6.81315, *MAPE_February_* = 0.214755, *MAPE_March_* = 0.474466, *MAPE_April_* = 0.117225, *MAPE_May_* = 0.105518, *MAPE_June_* = 0.0354902, *MAPE_July_* = 0.0251916, *MAPE_August_* = 0.0234608, *MAPE_September_* = 0.0352714, *MAPE_October_* = 0.0774352, *MAPE_November_* = 0.0570842, *MAPE_December_* = 0.0187244, and *MAPE_Total_* = 0.733775.

**Figure 5:**
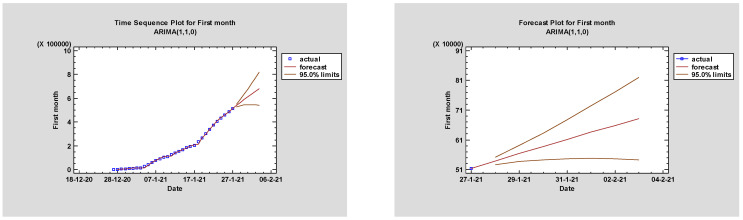

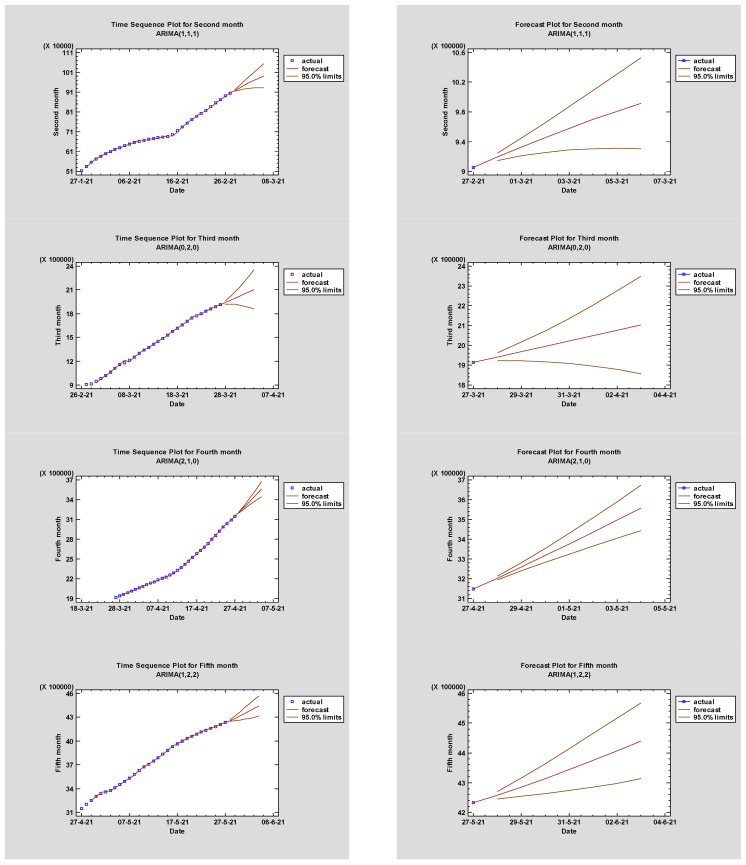

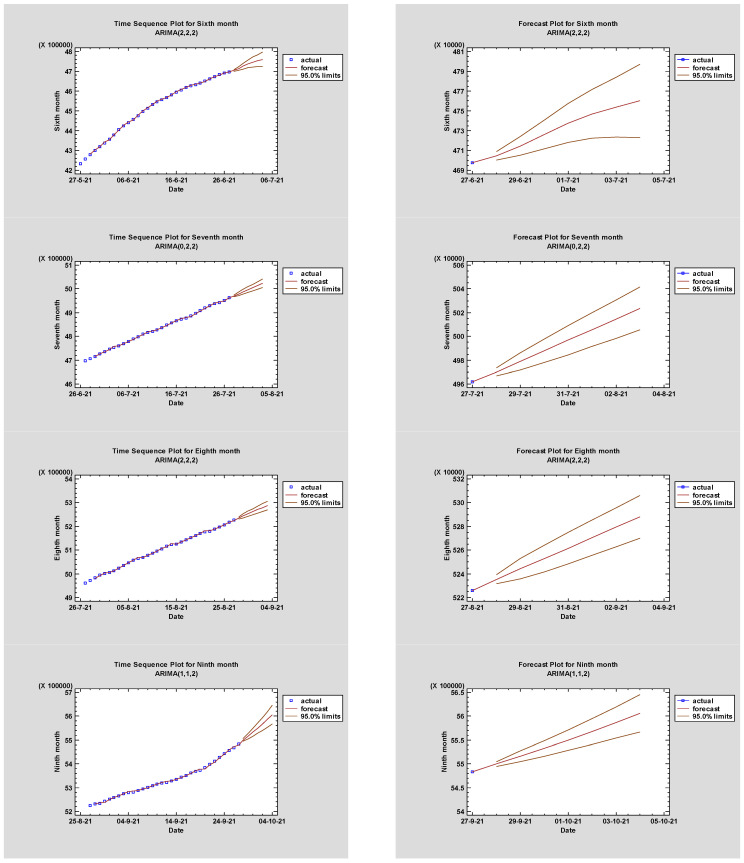

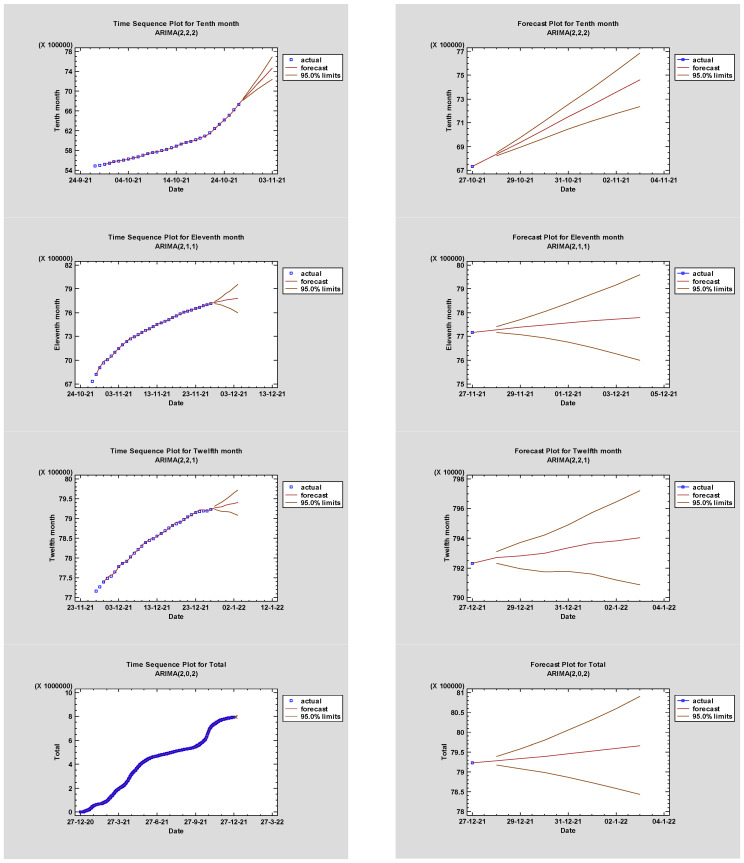
Forecast plots for ARIMA models in the next week per month and cumulative. In Figure 5 (left and right) are displayed the plots of the observed and forecasted maximized values for all twelve months and in total at 95.0% prediction limit for each forecast. Limits presented indicate the true value of each month and total at any point in future with likely to be with 95.0% confidence.

Table 2 highlights the parameter estimates for the best models with CI95% and *p*-values < 0.05, which were further confirmed through the Ljung–Box test. The forecasted and fitted values are detailed in Table 3 and Figure 5 for the next 7 days. Thus, the forecasts for the next week may be between 538,694–681,206. in January, 919,209–991,628. in February, 1.94174 × 10^6^–2.10285 × 10^6^ in March, 3.20358 × 10^6^–3.55743 × 10^6^ in April, 4.25759 × 10^6^–4.44011 × 10^6^ in May, 4.70459 × 10^6^–4.75999 × 10^6^ in June, 4.97002 × 10^6^–5.02345 × 10^6^ in July, 5.23552 × 10^6^–5.28803 × 10^6^ in August, 5.49969 × 10^6^–5.60622 × 10^6^ in September, 6.8339 × 10^6^–7.46267 × 10^6^ in October, 7.72849 × 10^6^–7.77887 × 10^6^ in November, 7.92705 × 10^6^–7.94037 × 10^6^ in December, and between 7.92819 × 10^6^–7.96656 × 10^6^ in Total.

## 4. Discussion

Ad26.COV2-S from *Johnson & Johnson* and BNT162b1/BNT162b2 from *Pfizer–BioNTech* were the top two opted vaccines against COVID-19 in Romania with over >50,000 individuals immunized (57,359 unique dose vs. 55,643 with dose 1 and 54,757 with dose 2), excepting the third dose (35,425 with the booster). mRNA-1273 by *Moderna* and AZD1222 by *AstraZeneca* were the least favorite (8501 with dose 1, 8225 with dose 2, 21,951 booster vs. 11,284 with dose 1, 10,685 with dose 2, and 9 with the booster). These results are further consolidated by the number of individuals who had a complete vaccination scheme and adverse reactions, both local and general; BNT162b1/BNT162b2 = 5,058,908 (1581 vs. 8451), Ad26.COV2-S = 1,913,978 (96 vs. 1122), mRNA-1273 = 399,327 (332 vs. 1936), and AZD1222 = 419.037 (138 vs. 6033).

As per ARIMA generated, the optimal models through the Ljung–Box Test are: ARIMA(1,1,0), ARIMA(1,1,1), ARIMA(0,2,0), ARIMA(2,1,0), ARIMA(1,2,2), ARIMA(2,2,2), ARIMA(0,2,2), ARIMA(2,2,2), ARIMA(1,1,2), ARIMA(2,2,2), ARIMA(2,1,1), ARIMA(2,2,1), and ARIMA (2,0,2) for all twelve months and in total.

The most recent article with a similar design is that of Cihan [28]. With over four hundred million people forecasted to be fully vaccinated by June 2021, the figures per hundred million were as follows: 147 in Europe (17%), 139 in Asia (2.3%), 130 in South America (8.8%), 129 in the US (41.8%), and 109 in Africa (0.6%) and 5.6% of the World. The optimal ARIMA models through the Ljung–Box test are ARIMA (5,2,2) in the US, ARIMA (1,2,3) in Asia, ARIMA (5,2,0) in Europe, ARIMA (2,2,1) in Africa, ARIMA (1,2,1) in South America, and ARIMA (5,2,1) in the World.

There will be an increased interest in vaccines between 5–10% in the first quarter of 2022. The short-term forecasts apply for influenza, HPV, pneumococcal, and polio vaccines, without being detected a decline in the overall interest for the COVID-19 vaccine [29]. However, confinement is still one of the most suitable prevention measures. The number of pediatric consultations/antenatal visits decreased by 52%/45% in April and 34%/34% in May 2020 compared to the same periods of 2019 (*p* = 0.0001), and demand for immunization significantly decreased as well [30].

Another nationwide article conducted is by Lumbreras-Marquez et al. [31], in which they briefly discuss the risk of morbidity and mortality among Mexican pregnant women. With 934 deaths in 2020, the maternal mortality ratio (MMR) was 46.6 per 100.00 live births, with 202 attributed to COVID-19. Around 31% (286/934) was associated with respiratory failure in contrast to 5% between 2011–2019 since the Mexican government launched the vaccination program on 11 May 2021. Assuming 100% vaccination among women, the authors forecasted weekly maternal deaths that might occur and obtained 993 deaths with an MMR of 46.5; RMSE (0,1,1) was 5.57 and 6.15 in 2021 compared to 2020 (21.6%). The overall figures would decrease to 885 and to an MMR of 41.5.

Distinct authors employed other mathematical models to perform predictive analyses in various countries [32,33]. Hwang [32] adopted a heterogeneous autoregression (HAR) model due to the long-memory feature of COVID-19 based on the growth and vaccination rates. Three novel perspectives derive from this protocol. The first refers to the combination of both growth and vaccination rates, construction and comparison of three types of predictions and coverage probability improvement, and mean interval score of prediction periods via bootstrap technique.

The Susceptible–Infected–Recovered (SIR) model [34] fits within the scope, with an expected effective reproduction term (*tR*) less than 1. According to a recent article, the tR reduces at fast rates when the values of *tR* are high, the slope being dependent on the promptness response parameter.

A multinomial autoregressive model for time series of counts was introduced with the aim of analyzing the finite-range integer-valued data. For this, the estimations of the parameters were calculated using conditional least squares (CLS), weighted conditional least squares (WCLS), and conditional maximum likelihood (CML). The authors established the asymptotic properties of the estimators and performed simulation studies to certify the current procedure [35]. Bartolucci et al. [36] and proposed multinomial Bayesian and Dirichlet auto-regressive models for series of time-dependent data points centered on counting patients exclusive and exhaustive categorized on predefined groups. Specifically, they were allocated based on the severity and required treatments in either regular wards or intensive care units, along with individuals that passed away and went through the disease. Not only were formulated assumptions on the transition probabilities between categories over a specific period that previously had a normal distribution allowed, but the accessibility to incorporate hypotheses was offered. Markov chain Monte Carlo (MCMC) was employed to estimate the posterior distribution and transition matrices, also allowing to make predictions and compute the reproduction number (*R*t), accuracy measured through Bayesian inference. In this way, the authors offer insight regarding data collection during the first wave in Lombardia, Italy, and the effect of non-pharmaceutical interventions. Furthermore, the Dirichlet-multinomial model is adequate in fitting/providing predictive performance for patients admitted in regular and intensive care units.

## 5. Conclusions

Our results emphasize the willingness of Romanian residents to get the vaccine. On the opposed pole, there is a significant discrepancy between the internal administration of Westernized countries with >50% of the overall population vaccinated and post-communist middle-class country such as Romania. Approximately 16 million doses have been administered since inception on 27 December 2020. BNT162b1/BNT162b2 and Ad26.COV2-S were the top two choices among the Romanian citizens, with figures comparable in contrast with mRNA-1273 and AZD1222 among all analyzed parameters. Statistical models still play a crucial role in making different predictions. As opposed to the existing literature, our study was focused on forecasting the vaccination rate, and not for establishing the infections trends. Following the analyses performed, ARIMA(1,1,0), ARIMA(1,1,1), ARIMA(0,2,0), ARIMA(2,1,0), ARIMA(1,2,2), ARI-MA(2,2,2), ARIMA(0,2,2), ARIMA(2,2,2), ARIMA(1,1,2), ARIMA(2,2,2), ARIMA(2,1,1), ARIMA(2,2,1), and ARIMA (2,0,2) are the best models that fit within the current situation from all scenarios generated based on their MAPE, *p*-value and through the Ljung–Box test. Conclusively, mathematical and statistical algorithms proved efficient in providing forecasts of either infectious or, in our case, vaccination trends in a country.

## Figures and Tables

**Table 1:** ARIMA models comparison.

Month	Model	RMSE	MAE	MAPE
January	ARIMA(1,1,0)	5983.61	3469.22	6.81315
	ARIMA(2,1,1)	6193.02	3478.39	6.81429
	ARIMA(2,1,0)	6074.41	3529.04	6.8465
	ARIMA(0,2,0)	5993.82	3589.63	6.98607
February	ARIMA(1,1,1)	2443.14	1496.05	0.214755
	ARIMA(1,1,2)	2404.11	1496.8	0.212518
	ARIMA(2,1,2)	2449.65	1497.25	0.212571
	ARIMA(2,1,0)	2366.97	1498.86	0.212441
	ARIMA(2,1,1)	2409.78	1500.66	0.212628
March	ARIMA(0,2,0)	10,125.3	6020.7	0.474466
	ARIMA(1,1,0)	10,069.7	6038.5	0.479113
	ARIMA(1,1,1)	9725.72	6091.12	0.500062
	ARIMA(1,2,0)	9870.77	6138.27	0.490985
	ARIMA(2,1,0)	9901.43	6183.01	0.50242
April	ARIMA(2,1,0)	4506.49	2856.4	0.117225
	ARIMA(1,1,0)	4429.84	2860.85	0.117355
	ARIMA(1,1,1)	4506.19	2864.04	0.117446
	ARIMA(2,1,1)	4582.38	2872.9	0.117986
	ARIMA(2,1,2)	4645.91	2896.7	0.119043
May	ARIMA(1,2,2)	5701.78	3844.24	0.105518
	ARIMA(2,2,2)	5817.73	3852.44	0.105606
	ARIMA(2,2,0)	6058.59	4005.54	0.110237
June	ARIMA(2,2,2)	2058.41	1593.3	0.0354902
	ARIMA(2,2,1)	2268.92	1694.63	0.0377449
	ARIMA(1,2,2)	2406.98	1741.78	0.0388305
	ARIMA(0,2,2)	2401.52	1784.57	0.0397096
July	ARIMA(0,2,2)	1610.49	1222.21	0.0251916
	ARIMA(2,2,2)	1633	1233.64	0.0254273
	ARIMA(1,2,2)	1634.18	1236.74	0.0254953
	ARIMA(2,1,2)	1986.95	1476.09	0.0304824
	ARIMA(1,1,2)	1949.75	1497.82	0.0309203
August	ARIMA(2,2,2)	1760.73	1196.04	0.0234608
	ARIMA(1,2,2)	1750.49	1217.35	0.023894
	ARIMA(0,2,2)	1719.34	1220.36	0.0239556
	ARIMA(1,2,1)	2142.33	1372.74	0.0269142
	ARIMA(2,1,2)	2216.94	1594.14	0.0313006
September	ARIMA(1,1,2)	2641.24	1891.2	0.0352714
	ARIMA(2,1,2)	2695.21	1929.22	0.0359849
	ARIMA(2,2,2)	2674.02	1981.65	0.0369697
	ARIMA(0,2,2)	2746.87	2089.46	0.0389583
	ARIMA(1,2,2)	2747.8	2096.01	0.0390938
October	ARIMA(2,2,2)	6287.29	4643.42	0.0774352
	ARIMA(2,2,1)	6897.6	5108.31	0.0847775
	ARIMA(1,2,2)	7032.4	5193.96	0.085918
	ARIMA(1,2,1)	6908.67	5262.54	0.0870042
November	ARIMA(2,1,1)	6121.27	4144.73	0.0570842
	ARIMA(2,1,0)	5986.3	4187.46	0.0577338
	ARIMA(2,1,2)	6207.1	4200.65	0.0579711
December	ARIMA(2,2,1)	1891.64	1470.03	0.0187244
	ARIMA(2,2,0)	1856.3	1473.05	0.0187625
	ARIMA(2,2,2)	1916.62	1499.57	0.0191095
	ARIMA(0,2,2)	2016.04	1643.22	0.0209753
Total	ARIMA(2,0,2)	5360.97	3259.29	0.733775
	ARIMA(2,2,0)	5394.91	3268.17	0.695145
	ARIMA(2,2,1)	5399.27	3268.9	0.695924
	ARIMA(2,2,2)	5406.99	3271.66	0.696387
	ARIMA(1,1,1)	5393.81	3274.41	0.688878

**Table 2:** ARIMA models parameters.

Month	Parameter	Estimate	Standard Error	t-Statistic	*p*-Value	Ljung–Box Test
January	AR(1)	0.982382	0.0548607	17.9069	0	0.102632
February	AR(1)	0.9564	0.0333551	28.6733	0	0.864548
	MA(1)	−0.168594	0.118083	−1.42776	0.164042	
March	no parameter(s)					0.477973
April	AR(1)	1.03694	0.18565	5.58548	0.000005	0.248501
	AR(2)	−0.0224815	0.189402	−0.118698	0.906333	
May	AR(1)	0.759005	0.158601	4.78564	0.000059	0.986002
	MA(1)	0.431691	0.165075	2.61512	0.01465	
	MA(2)	0.575053	0.156229	3.68084	0.001069	
June	AR(1)	1.18927	0.0786618	15.1188	0	0.0169788
	AR(2)	−0.975677	0.0728746	−13.3884	0	
	MA(1)	1.26288	0.159909	7.89746	0	
	MA(2)	−0.808264	0.123585	−6.54012	0.000001	
July	MA(1)	0.0470354	0.0932973	0.504145	0.618249	0.0043751
	MA(2)	0.868584	0.0876427	9.91051	0	
August	AR(1)	0.0639607	0.207405	0.308385	0.760246	0.000105
	AR(2)	−0.186658	0.196426	−0.950269	0.350726	
	MA(1)	0.0050157	0.0645373	0.0777177	0.938648	
	MA(2)	0.95	0.053573	17.7328	0	
September	AR(1)	1.03825	0.0200273	51.8419	0	0.0136246
	MA(1)	0.29945	0.190727	1.57004	0.127639	
	MA(2)	0.371627	0.175248	2.12057	0.042949	
October	AR(1)	−0.211891	0.182648	−1.16011	0.256963	0.690025
	AR(2)	−0.702692	0.168935	−4.15955	0.000329	
	MA(1)	−1.00778	0.157958	−6.38005	0.000001	
	MA(2)	−0.780661	0.166924	−4.67675	0.000086	
November	AR(1)	1.49501	0.212479	7.03601	0	0.0059234
	AR(2)	−0.530011	0.211406	−2.50708	0.018252	
	MA(1)	0.267886	0.318328	0.841539	0.407176	
December	AR(1)	−0.0584976	0.202084	−0.289471	0.774516	0.175271
	AR(2)	−0.745858	0.135203	−5.51656	0.000009	
	MA(1)	−0.0445405	0.265186	−0.167959	0.867915	
Total	AR(1)	1.97226	0.0144948	136.067	0	1.11 × 10^−16^
	AR(2)	−0.972185	0.0145503	−66.8156	0	
	MA(1)	−0.147522	0.0546424	−2.69977	0.007264	
	MA(2)	0.103778	0.0542596	1.91261	0.056587	

**Table 3:** Prediction of vaccinated individuals against COVID-19 per month and total for the next week according to our ARIMA with CI95%.

		Lower 95%	Upper 95%
Period	Forecast	Limit	Limit
**January**			
28 January 2021	538,694	526,474	550,914
29 January 2021	563,514	536,381	590,647
30 January 2021	587,896	542,802	632,991
31 January 2021	611,849	546,277	677,421
1 February 2021	635,380	547,182	723,579
2 February 2021	658,497	545,792	771,201
3 February 2021	681,206	542,325	820,086
**February**			
28 February 2021	919,209	914,179	924,239
1 March 2021	932,663	920,849	944,477
2 March 2021	945,530	925,560	965,501
3 March 2021	957,836	928,655	987,018
4 March 2021	969,606	930,370	1.00884 × 10^6^
5 March 2021	980,862	930,880	1.03084 × 10^6^
6 March 2021	991,628	930,327	1.05293 × 10^6^
**March**			
28 March 2021	1.94174 × 10^6^	1.92096 × 10^6^	1.96251 × 10^6^
29 March 2021	1.96859 × 10^6^	1.92213 × 10^6^	2.01504 × 10^6^
30 March 2021	1.99544 × 10^6^	1.91771 × 10^6^	2.07317 × 10^6^
31 March 2021	2.02229 × 10^6^	1.9085 × 10^6^	2.13608 × 10^6^
1 April 2021	2.04914 × 10^6^	1.89507 × 10^6^	2.20322 × 10^6^
2 April 2021	2.076 × 10^6^	1.87781 × 10^6^	2.27418 × 10^6^
3 April 2021	2.10285 × 10^6^	1.85703 × 10^6^	2.34867 × 10^6^
**April**			
28 April 2021	3.20358 × 10^6^	3.19435 × 10^6^	3.2128 × 10^6^
29 April 2021	3.26041 × 10^6^	3.23948 × 10^6^	3.28134 × 10^6^
30 April 2021	3.31808 × 10^6^	3.28272 × 10^6^	3.35345 × 10^6^
1 May 2021	3.37661 × 10^6^	3.32444 × 10^6^	3.42878 × 10^6^
2 May 2021	3.436 × 10^6^	3.36486 × 10^6^	3.50714 × 10^6^
3 May 2021	3.49627 × 10^6^	3.40416 × 10^6^	3.58838 × 10^6^
4 May 2021	3.55743 × 10^6^	3.44246 × 10^6^	3.6724 × 10^6^
**May**			
28 May 2021	4.25759 × 10^6^	4.24549 × 10^6^	4.26969 × 10^6^
29 May 2021	4.28472 × 10^6^	4.25408 × 10^6^	4.31536 × 10^6^
30 May 2021	4.31365 × 10^6^	4.26305 × 10^6^	4.36424 × 10^6^
31 May 2021	4.34394 × 10^6^	4.27326 × 10^6^	4.41462 × 10^6^
1 June 2021	4.37527 × 10^6^	4.28497 × 10^6^	4.46557 × 10^6^
2 June 2021	4.40739 × 10^6^	4.29825 × 10^6^	4.51653 × 10^6^
3 June 2021	4.44011 × 10^6^	4.31303 × 10^6^	4.56718 × 10^6^
**June**			
28 June 2021	4.70459 × 10^6^	4.70025 × 10^6^	4.70894 × 10^6^
29 June 2021	4.71453 × 10^6^	4.70509 × 10^6^	4.72396 × 10^6^
30 June 2021	4.72637 × 10^6^	4.71166 × 10^6^	4.74108 × 10^6^
1 July 2021	4.73785 × 10^6^	4.71808 × 10^6^	4.75761 × 10^6^
2 July 2021	4.74701 × 10^6^	4.7222 × 10^6^	4.77182 × 10^6^
3 July 2021	4.75379 × 10^6^	4.72335 × 10^6^	4.78424 × 10^6^
4 July 2021	4.75999 × 10^6^	4.72278 × 10^6^	4.79721 × 10^6^
**July**			
28 July 2021	4.97002 × 10^6^	4.9667 × 10^6^	4.97334 × 10^6^
29 July 2021	4.97892 × 10^6^	4.97164 × 10^6^	4.98621 × 10^6^
30 July 2021	4.98783 × 10^6^	4.97788 × 10^6^	4.99777 × 10^6^
31 July 2021	4.99673 × 10^6^	4.98455 × 10^6^	5.00892 × 10^6^
1 August 2021	5.00564 × 10^6^	4.99142 × 10^6^	5.01986 × 10^6^
2 August 2021	5.01454 × 10^6^	4.99842 × 10^6^	5.03067 × 10^6^
3 August 2021	5.02345 × 10^6^	5.0055 × 10^6^	5.0414 × 10^6^
**August**			
28 August 2021	5.23552 × 10^6^	5.2318 × 10^6^	5.23923 × 10^6^
29 August 2021	5.24423 × 10^6^	5.23572 × 10^6^	5.25274 × 10^6^
30 August 2021	5.25285 × 10^6^	5.24159 × 10^6^	5.26412 × 10^6^
31 August 2021	5.26164 × 10^6^	5.24849 × 10^6^	5.2748 × 10^6^
1 September 2021	5.27046 × 10^6^	5.25557 × 10^6^	5.28535 × 10^6^
2 September 2021	5.27925 × 10^6^	5.26269 × 10^6^	5.29582 × 10^6^
3 September 2021	5.28803 × 10^6^	5.26988 × 10^6^	5.30618 × 10^6^
**September**			
28 September 2021	5.49969 × 10^6^	5.49415 × 10^6^	5.50524 × 10^6^
29 September 2021	5.51582 × 10^6^	5.5047 × 10^6^	5.52695 × 10^6^
30 September 2021	5.53257 × 10^6^	5.51633 × 10^6^	5.54881 × 10^6^
1 October 2021	5.54996 × 10^6^	5.52844 × 10^6^	5.57148 × 10^6^
2 October 2021	5.56801 × 10^6^	5.54092 × 10^6^	5.59511 × 10^6^
3 October 2021	5.58676 × 10^6^	5.5537 × 10^6^	5.61982 × 10^6^
4 October 2021	5.60622 × 10^6^	5.5668 × 10^6^	5.64564 × 10^6^
**October**			
28 October 2021	6.8339 × 10^6^	6.82067 × 10^6^	6.84713 × 10^6^
29 October 2021	6.93473 × 10^6^	6.89546 × 10^6^	6.97401 × 10^6^
30 October 2021	7.04299 × 10^6^	6.97166 × 10^6^	7.11432 × 10^6^
31 October 2021	7.14982 × 10^6^	7.04635 × 10^6^	7.25329 × 10^6^
1 November 2021	7.25173 × 10^6^	7.1128 × 10^6^	7.39067 × 10^6^
2 November 2021	7.35569 × 10^6^	7.17533 × 10^6^	7.53606 × 10^6^
3 November 2021	7.46267 × 10^6^	7.23757 × 10^6^	7.68778 × 10^6^
**November**			
28 November 2021	7.72849 × 10^6^	7.71583 × 10^6^	7.74114 × 10^6^
29 November 2021	7.73911 × 10^6^	7.70822 × 10^6^	7.77 × 10^6^
30 November 2021	7.74866 × 10^6^	7.69434 × 10^6^	7.80297 × 10^6^
1 December 2021	7.7573 × 10^6^	7.67552 × 10^6^	7.83907 × 10^6^
2 December 2021	7.76515 × 10^6^	7.65286 × 10^6^	7.87745 × 10^6^
3 December 2021	7.77232 × 10^6^	7.6272 × 10^6^	7.91745 × 10^6^
4 December 2021	7.77887 × 10^6^	7.59921 × 10^6^	7.95853 × 10^6^
**December**			
28 December 2021	7.92705 × 10^6^	7.92313 × 10^6^	7.93097 × 10^6^
29 December 2021	7.92826 × 10^6^	7.91954 × 10^6^	7.93697 × 10^6^
30 December 2021	7.92976 × 10^6^	7.91742 × 10^6^	7.9421 × 10^6^
31 December 2021	7.93335 × 10^6^	7.91754 × 10^6^	7.94916 × 10^6^
1 January 2022	7.9366 × 10^6^	7.91599 × 10^6^	7.9572 × 10^6^
2 January 2022	7.93831 × 10^6^	7.91207 × 10^6^	7.96455 × 10^6^
3 January 2022	7.94037 × 10^6^	7.90872 × 10^6^	7.97201 × 10^6^
**Total**			
28 December 2021	7.92819 × 10^6^	7.91765 × 10^6^	7.93873 × 10^6^
29 December 2021	7.9335 × 10^6^	7.90879 × 10^6^	7.95822 × 10^6^
30 December 2021	7.93926 × 10^6^	7.89825 × 10^6^	7.98028 × 10^6^
31 December 2021	7.94546 × 10^6^	7.88616 × 10^6^	8.00477 × 10^6^
1 January 2022	7.95208 × 10^6^	7.87278 × 10^6^	8.03139 × 10^6^
2 January 2022	7.95912 × 10^6^	7.85832 × 10^6^	8.05991 × 10^6^
3 January 2022	7.96656 × 10^6^	7.84298 × 10^6^	8.09014 × 10^6^

## Data Availability

The datasets used and analyzed during the current study are available from the corresponding author on reasonable request.
